# Pre-implantation developmental potential from in vivo and in vitro matured mouse oocytes: a cytoskeletal perspective on oocyte quality

**DOI:** 10.1007/s10815-014-0363-4

**Published:** 2014-11-09

**Authors:** Alexandra Sanfins, Carlos E. Plancha, David F. Albertini

**Affiliations:** 1 Faculdade de Medicina Veterinária, Universidade Lusófona de Humanidades e Tecnologias, Campo Grande 376, 1749-024 Lisbon, Portugal; 2 Unidade de Biologia da Reprodução, Instituto de Histologia e Biologia do Desenvolvimento, Faculdade de Medicina da Universidade de Lisboa, 1649-028 Lisbon, Portugal; 3 Centro Médico de Assistência à Reprodução - CEMEARE, Rua Alfredo Mesquita, 2E, 1600-922 Lisbon, Portugal; 4 Department of Molecular and Integrative Physiology, University of Kansas Medical Center, 3901 Rainbow Boulevard, Kansas City, KS 66160-7401 USA

**Keywords:** Oocyte maturation, Blastocyst, Cell cycle arrest, Roscovitine, Blastomere

## Abstract

**Purpose:**

In the present study, fertilization and developmental potential of mouse oocytes matured in different conditions were tested. The efficiency of in vitro fertilization (IVF), pre-implantation development and some important aspects of cytokinesis during early cleavages are discussed.

**Methods:**

In vivo matured (IVO), in vitro matured (IVM) and roscovitine-treated (IVM-Rosco) mouse oocytes were subjected to IVF under identical conditions. Three replicates per group were analyzed. Fertilization was identified by the presence of two pronuclei at 6–8 h post-fertilization. Evaluation of pre-implantation embryonic development was done daily from day 2 to day 5 and embryos were processed for analyses of chromatin, nuclear lamina, microtubules and centrosomal proteins by conventional and confocal fluorescence microscopy.

**Results:**

Both IVM groups displayed lower fertilization rates when compared to in vivo controls. While IVO-derived embryos exhibit efficient and synchronous progression to the blastocyst stage, both IVM-derived embryos exhibit a delay in embryonic progression, and a lower blastocyst rate. Interestingly, IVM-Rosco M-II oocytes exhibited more blastomere symmetries and higher number of cells at the blastocyst stage than the IVM group with the most notable influence being on the centrosome-microtubule complex of blastomeres.

**Conclusion:**

Our study strongly indicates that when compared to spontaneously in vitro matured oocytes, treatment with roscovitine may partially enhance developmental competence by maintaining coordination between nuclear and cytoplasmic events. Further evidence is given of cytoskeletal biomarkers that can be identified during in vitro oocyte maturation conditions.

## Introduction

Oocyte maturation is the final phase of oogenesis during which a number of complex changes take place that involve progression of the oocyte from prophase-I to metaphase-II [1, 2]. This process involves coordination of nuclear and cytoplasmic events necessary to produce quality oocytes capable of being fertilized and support preimplantation development [3]. In vivo, this process depends on the close interaction between the cumulus cells and the oocyte involving a cascade of signaling pathways, particularly EGF-like proteins produced from the cumulus cells in response to LH [4]. Since the original experiments of Pincus & Enzman (1935) and Edwards (1965) it became clear that oocytes undergo immediate and spontaneous maturation once removed from their follicular environment [5, 6]. However, while IVM supports nuclear maturation, cytoplasmic maturation, a process required for the expression of developmental competence, is not assured [3]. Overcoming the obstacles limiting cytoplasmic maturation during IVM remains a challenge for the production of quality oocytes of clinical value to the practice of human assisted reproductive technologies (ARTs) [7]. Several features have been described that might contribute to the high developmental competence of IVO oocytes when compared to their IVM counterparts, such as ATP concentration [8], number and distribution of active mitochondria [9], glutathione content [10], transcriptional and translational activity [11, 12], and imprinting/methylation patterns in embryos derived from IVO and IVM [13]. Our group has been especially interested in analyzing patterns of cytoskeletal organization in IVO and IVM matured oocytes to better define properties indicative of developmental competence. Specifically, when spindle size / shape and distribution of cytoplasmic Microtubule Organizing Centers (MTOCs) were analyzed, IVO oocytes exhibited compact and pointed-shaped spindles with increased number of cytoplasmic MTOCs. In contrast, IVM oocytes displayed barrel-shaped spindles, which exhibited much more γ-tubulin aggregates at the spindle poles, associated with a low number of cytoplasmic MTOCs [14]. These features were found to result from the distinct cell cycle regulation with differential allocation of γ-tubulin stores during the first 5 h of oocyte maturation [15]. Thus, spindle morphogenesis in IVM oocytes favors recruitment of γ-tubulin to the spindle, while retention of γ-tubulin foci in the cytoplasm with reduced γ-tubulin recruitment to the spindle is observed in IVO oocytes. Consequently, depletion of maternal factors, like γ-tubulin, from the cytoplasm is observed upon IVM and expected to increase with emission of the second polar body after fertilization [16–18]. These critical differences in cytoskeleton organization were found to be a result of the distinct cell cycle progression between IVO and IVM oocytes. In fact, in IVO oocytes the follicular environment maintains the G2/M cell cycle delay necessary to keep nuclear lamina integrity and proper localization of cytoplasmic MTOCs which is not observed under IVM conditions [15].

The communication established between the oocyte and its surrounding cumulus cells is crucial to promote the oocyte nuclear and cytoplasmic maturation needed to achieve developmental competence [17–20]. When a G2/M phase delay was imposed in culture by treating oocytes with the specific MPF inhibitor, roscovitine, IVO-like cytoskeletal characteristics were partially achieved in IVM oocytes (IVM-Rosco), namely pointed-shape and cortically localized spindles associated with an increased number of cytoplasmic MTOCs [15] suggesting that an imposed delay in cell cycle progression may be necessary to allow nuclear changes to wait for the cytoplasmic maturation events.

In the present study, additional parameters of oocyte quality, in particular fertilization and developmental competencies are evaluated after careful comparison of the pre-implantation development characteristics between embryo-derived IVO, IVM and IVM-Rosco oocytes. Special emphasis is placed on the expression of cytoskeletal proteins involved in cell polarity determination and stability of the nucleus.

## Material and methods

### Animals

All oocytes were obtained from CF-1 outbred mice (Harlan-Sprague Dawley, Indianapolis, IN) between 6 and 8 week of age. Animals were handled according to the *Guide for Care and Use of Laboratory Animals* (National Academy of Science, 1996) and maintained on a 14L:10D photoperiod under constant temperature and relative humidity conditions. Food and water were provided ad libitum. Five females were kept per cage and a total of 50 females were used for this study. For IVF and mating experiments sperm was obtained from B6D2F1 hybrid male mice (Harlan-Sprague Dawley, Indianapolis, IN) between 8 and 12 week-old. Sexually matured males of proven fertility were used after successful mating and after abstaining for 1 week.

### Collection and culture of oocytes

For all experiments three types of oocyte collections were preformed with either IVO oocytes or IVM oocytes. IVO oocytes were obtained from mice previously injected 46 h earlier with 5 IU of equine chorionic gonadotropin (eCG, CalBiochem) to stimulate follicular development followed 46 h later by 5 IU of human chorionic gonadotropin (hCG, Sigma). Oocytes were collected from the swollen ampulla 15 h post-hCG in collection medium consisting of Hepes-buffered Eagles MEM with Hanks’salts supplemented with 100 IU/ml penicillin, 100 μg/ml streptomycin and 0.3 % BSA. Only MII-stage oocytes were isolated.

IVM oocytes were obtained from mice injected 46 h earlier with 5 IU of eCG and cumulus-enclosed oocytes (COCs) were isolated by follicular puncture in collection medium. COCs were cultured for 15 h in in vitro maturation medium (IVM oocytes) consisting of Eagle’s MEM supplemented with Earle’s salts, 2 mM glutamine, 0.23 mM pyruvate, 100 IU/ml penicillin, 100 μg/ml streptomycin, 0.3 % BSA and 1 mg/ml fetuin (SIGMA) which had been previously dialyzed against MEM for 2 days using a slide-A-lyzer dialysis cassette (Pierce Biotechnology, Rockford, IL). Only MII-stage oocytes were isolated and used for these experiments. These oocytes will be referred as IVM oocytes. In parallel COCs were cultured in in vitro maturation medium containing 50 μM Roscovitine (BIOMOL, Pennsylvania, USA) for 3 h. Following roscovitine treatment, oocytes were washed three times in 100x volume of maturation medium without roscovitine and were subsequently allowed to mature in vitro for 15 h without drug as described above. Only MII-stage oocytes were isolated and used for these experiments. These oocytes will be referred as IVM-Rosco oocytes. All cultures were preformed in a humidified atmosphere of 5 % CO_2_ in air at 37 °C.

### In vitro fertilization and embryo culture

IVF and embryo culture were preformed as previously described only using M-II oocytes identified in each group [8]. IVO, IVM and IVM-Rosco oocytes were fertilized and embryos cultured under the same conditions. Additionally, pronuclear (PN) stage embryos were isolated upon visualization of vaginal plugs in females that received hCG and were mated with males of proven fertility. A total of 3 full experiments were performed. All culture media was prepared fresh using frozen reagents (Sigma Biosciences) kept at −20 °C for up to 2 months. Drops of media overlaid with filtered embryo-tested mineral oil (Sigma Biosciences) were set up the day before and allowed to equilibrate overnight in a humidified atmosphere of 5 % CO_2_ in air at 37 °C. Media composition is described elsewhere [21, 22].

Sperm was collected from caudae epididymes and placed in a 200 μl drop of modified Tyrode medium under oil in a 35 mm Falcon dish. Epididymal content was released using watchmaker’s forceps and allowed to swim out for 10 min. Subsequently, epididymes were removed and a 100 μl volume of this preparation was diluted 1:4 in modified Tyrode medium under oil for capacitation in 4 well-Nunc dishes. Sperm was allowed to capacitate for 90 min at 37 °C in a humidified atmosphere of 5 % CO_2_ in air. During this period sperm concentration was determined using a hemocytometer.

IVF was preformed using only M-II oocytes produced from the following groups: 1) M-II IVO oocytes collected from the ampulla at 15 h post-hCG; 2) M-II IVM and 3) M-II IVM-Rosco oocytes previously cultured for 15 h as described above. For collection of IVO oocytes oviducts were placed into Hepes-free PBS supplemented with 100 IU/ml penicillin and 100 μg/ml streptomycin as described [8]. A single oviduct was placed directly into the oil surrounding the 200-μl drop of modified potassium simplex optimization media (KSOM) [22]. Intact cumulus masses were released from the ampulla and gently placed into the drop of modified KSOM. This procedure allows the transfer of cumulus masses with the minimum amount of debris and PBS into the fertilization drop. Following collection of the cumulus masses, capacitated sperm was added to the fertilization drop at a final motile concentration of 800,000–900,000 sperm/ml. For fertilization of both IVM and IVM-Rosco oocytes, cumulus cells were removed by gentle pipetting and oocytes were extensively washed in 50 μl drops of modified KSOM media. Subsequently, oocytes were transfer with minimum amount of media into the 200 μl drop of modified KSOM and sperm added as above. Fertilization was carried out for 4 h at 37 °C in a humidified atmosphere of 5 % CO_2_ in air. 4 h later the eggs were washed several times in 50 μl drops of KSOM [21]. 10–15 zygotes were placed in 50 μl drops of KSOM under oil and cultured in a humidified atmosphere of 5 % CO_2_ at 37 °C. Fertilization was assessed by the presence of two PN 6–8 h post-insemination and confirmed by the development of 2-cell stage embryos the next day. For these studies day 1 was consider the day of fertilization. Evaluation of embryonic development (2-cell, 4-cell, 8-cell, compacted morula and blastocyst) was done on day 2, day 3, day 4 and day 5.

### Mating and embryo culture

As a control for these experiments, PN-stage embryos were collected from pregnant females and cultured as above. After hCG injection, 2 females were placed overnight with one male. The presence of a vaginal plug accessed the next morning was considered a successful mating. If so, males were separated and females were sacrificed 21 h post-hCG for collection of PN-stage embryos from the oviduct. 10 to 15 PN-stage embryos were placed in culture as described above.

### Processing of embryos for fluorescence microscopy

In addition to evaluation of embryonic development, control, IVO, IVM and IVM-Rosco-derived embryos were fixed at each day of development, and processed for constitutive (pericentrin) or regulative (γ-tubulin) MTOC markers, microtubules, chromatin and lamin B distribution as previously described [14]. Labeling pairs in sequence were (a) mouse monoclonal anti-γ-tubulin (1:100 dilution, Sigma) followed by Alexa-fluor 568 goat anti-mouse IgG (1:800 dilution, Molecular Probes). This sample was subsequently stained with rabbit polyclonal nuclear lamin B (1:200 dilution) [21, 23] followed by Alexa-fluor 488 goat anti-rabbit IgG (1:600 dilution, Molecular Probes); (b) polyclonal anti-pericentrin (1:200 dilution, Covance, CA) followed by Alexa-fluor 568 goat anti-rabbit IgG (1:800, Molecular Probes). This sample was subsequently stained with rat monoclonal to α-tubulin YOL-34 (1:200 dilution) [24] followed by Alexa-fluor 488 goat anti-rat IgG (1:600 dilution, Molecular Probes). Embryos were mounted in a non-compressed way using a mixture of vasolin:lanolin:parafin (1:1:1) that was placed on the edges of the coverslip. A 1.5 μl of a 50 % glycerol/PBS containing sodium azide and Hoechst 33258 (1 μg/ml Polysciences Inc., Warrington, PA) was used as mounting medium. Total cell counts at the blastocyst stage were determined based on the Hoechst 33258 staining of the samples produced. Labeled embryos were analyzed using a Zeiss IM-35 inverted microscope and a 50-W mercury arc lamp using 40x and 63x Neofluor objectives. Digital images were collected with a Hamamatsu Orca ER digital camera (model #C4742-95) interfaced with a Meta Morph Imaging System. A triple band pass dichroic and automated excitation filter selection permitted collection of in-frame images with minimal magnification or spatial distortion.

### Confocal microscopy

Triple labeled samples prepared as described above were analyzed by confocal microscopy using a Zeiss LSM Pascal system mounted on a Zeiss Axioscope II [63x plan Neofluor oil 1.4 numerical aperture (n.a.) or 40x water immersion objectives 1.2 n.a.]. LSM 5 Image Browser was used to analyze samples with complete Z-axis data sets of 30–50 sections per embryo at 0.5–0.7 μm depending on the embryonic stage. HeNe laser excitation for each probe permitted complete spatial restoration of Z-series or computed 3-D projections.

## Results

Cytoskeletal organization of IVO and IVM oocytes has been extensively analyzed in terms of their distinct spindle shape, size and MTOCs organization [14]. We have also shown that M-II IVM-Rosco oocytes displayed characteristics closer to their IVO counterparts reflecting pointed shaped spindles and increased number of cytoplasmic MTOCs [15]. Considering that these differences could be used as potential biomarkers of oocyte developmental potential, the present study aimed to carefully assess the pre-implantation development characteristics of embryo-derived in vitro matured oocytes.

### In vitro maturation impairs fertilization capacity

**Fig. 1:**
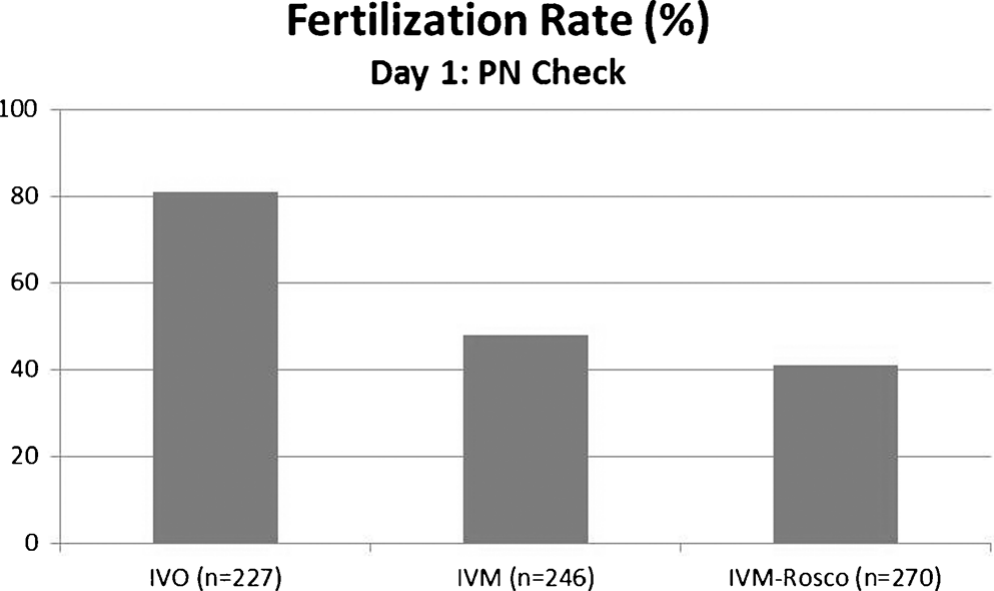
Fertilization efficiency checked by the presence of a visible PN 6–8 h post-fertilization of IVO, IVM and IVM-Rosco oocytes (81 % for IVO, 48 % for IVM, and 41 % for IVM-Rosco). N represents total number of embryos used

IVM and IVM-Rosco oocytes were cultured for 15 h and the efficiency of maturation was evaluated. As shown in Table 1, both protocols of in vitro maturation were not detrimental in terms of meiotic progression since high percentage of oocytes reached the M-II stage (82 % of IVM versus 78 % IVM-Rosco). Only M-II oocytes were used for IVF. Fertilization efficiency was assessed by the presence of two visible PN, 6 to 8 h post-fertilization. IVO, IVM and IVM-Rosco oocytes exhibit distinct IVF efficiencies as shown in Fig. 1. Only 48 % of PN-stage embryos were produced after IVF from IVM oocytes, and 41 % from IVM-Rosco oocytes, in contrast to the 81 % of IVO oocytes that resumed meiosis and formed PN.

**Table 1:** Maturation efficiency of IVM and IVM-Rosco oocytes based in two experiments. N represents total number of oocytes used

	GV (%)	M-I (%)	M-II (%)	Degenerated (%)
IVM (*N* = 100)	3.7	6.5	82.4	7.4
IVM-Rosco (*N* = 107)	1.8	11	78	9.2

### In vitro maturation affects developmental potential

**Fig. 2:**
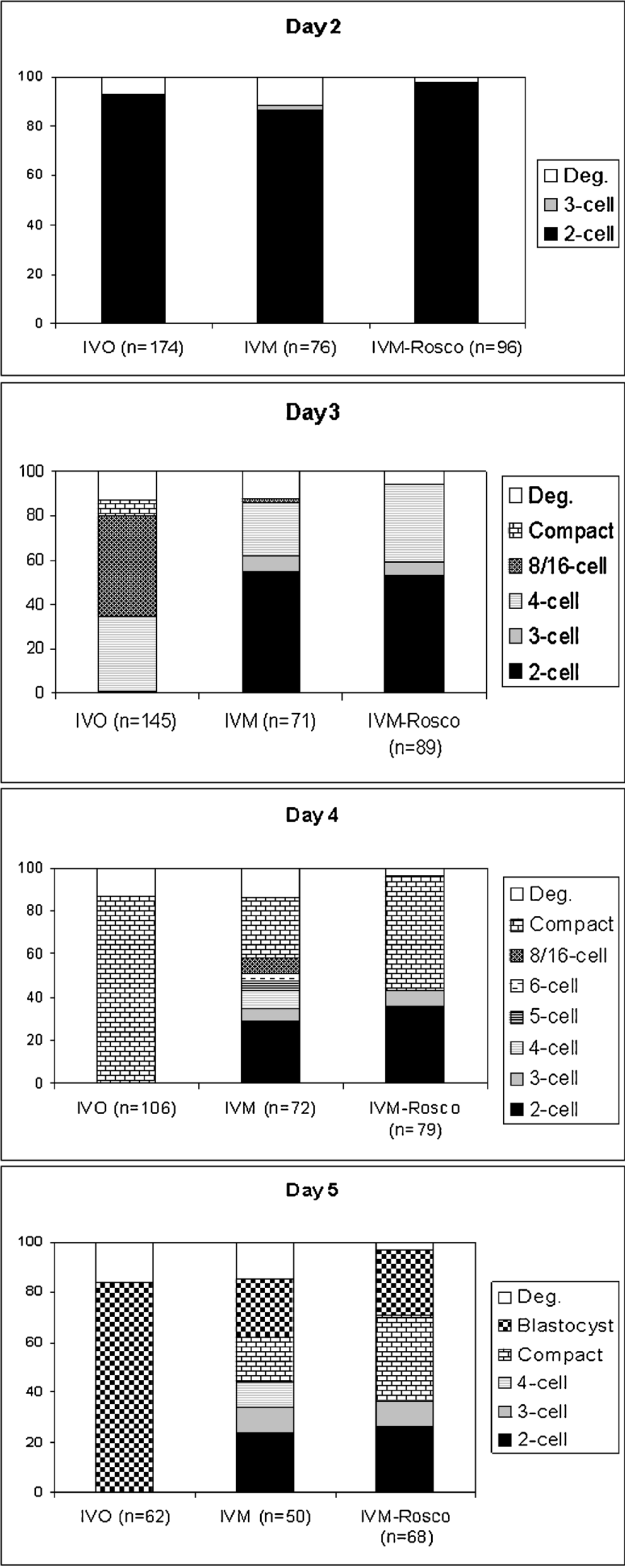
Development until the blastocyst stage of IVO, IVM and IVM-Rosco fertilized eggs. Note the synchronism of IVO oocytes contrasting with the asynchronous cleavages of IVM oocytes that exhibit even number of blastomeres on day 4 and day 5. The efficiency of blastocyst formation is higher in IVO embryos (84 %) when compared with IVM embryos (23 %). IVM-Rosco embryos exhibit an intermediate synchronism of cleavage rates when compared to both IVO and IVM embryos. The absence of even numbers of blastomeres in the IVM-Rosco group could be an indicator of proper cleavage rates (in contrast to what happen with IVM embryos). Also note the presence of 2-cell stage embryos throughout development in both IVM and IVM-Rosco oocytes

We next determined the pre-implantation development of the embryos produced by assessing daily their cleavage rates. Results are shown in Fig. 2.

The cleavage rate of IVM-derived embryos is distinct when compared to IVO-derived embryos: while IVO-derived embryos exhibit efficient and synchronous progression until the blastocyst stage, IVM-derived embryos exhibit a variable degree of delay in cleavage rate evident by the simultaneous presence of embryos at different stages of development, as shown in Fig. 2. Therefore, at day 4 while most of the embryos in the IVO group display the compacted morula stage (87 %), IVM-derived embryos exhibit a broader range of cleavage stages, namely 2-cell (29 %), 3-cell (5.6 %), 4-cell (8.3 %), 5-cell (4.1 %), 6-cell (4.1 %), 8-16-cell (7 %) and compacted morula (28 %). Interestingly, when compared to IVM-derived embryos, IVM-Rosco embryos do not display such a broad variability on embryonic stages throughout pre-implantation development suggesting a less delayed progression (on day 4: 35.4 % at 2-cell, 7.6 % at 3-cell, and 53.2 % compacted morula).

When comparing pre-implantation development of IVO and IVM-derived embryos at day 5 a dramatic decrease in the percentage of blastocysts produced is observed on both IVM and IVM-Rosco-derived embryos (84 % in IVO versus 23 % in IVM and 27 % in IVM-Rosco). Interestingly, as in the previous day, IVM-Rosco embryos seem to be more advanced than their IVM counterparts, presenting 40 % of compacted morula stage instead of only 20 %.

### In vitro maturation affects blastomere cleavage symmetry and nuclear morphology

**Fig. 3:**
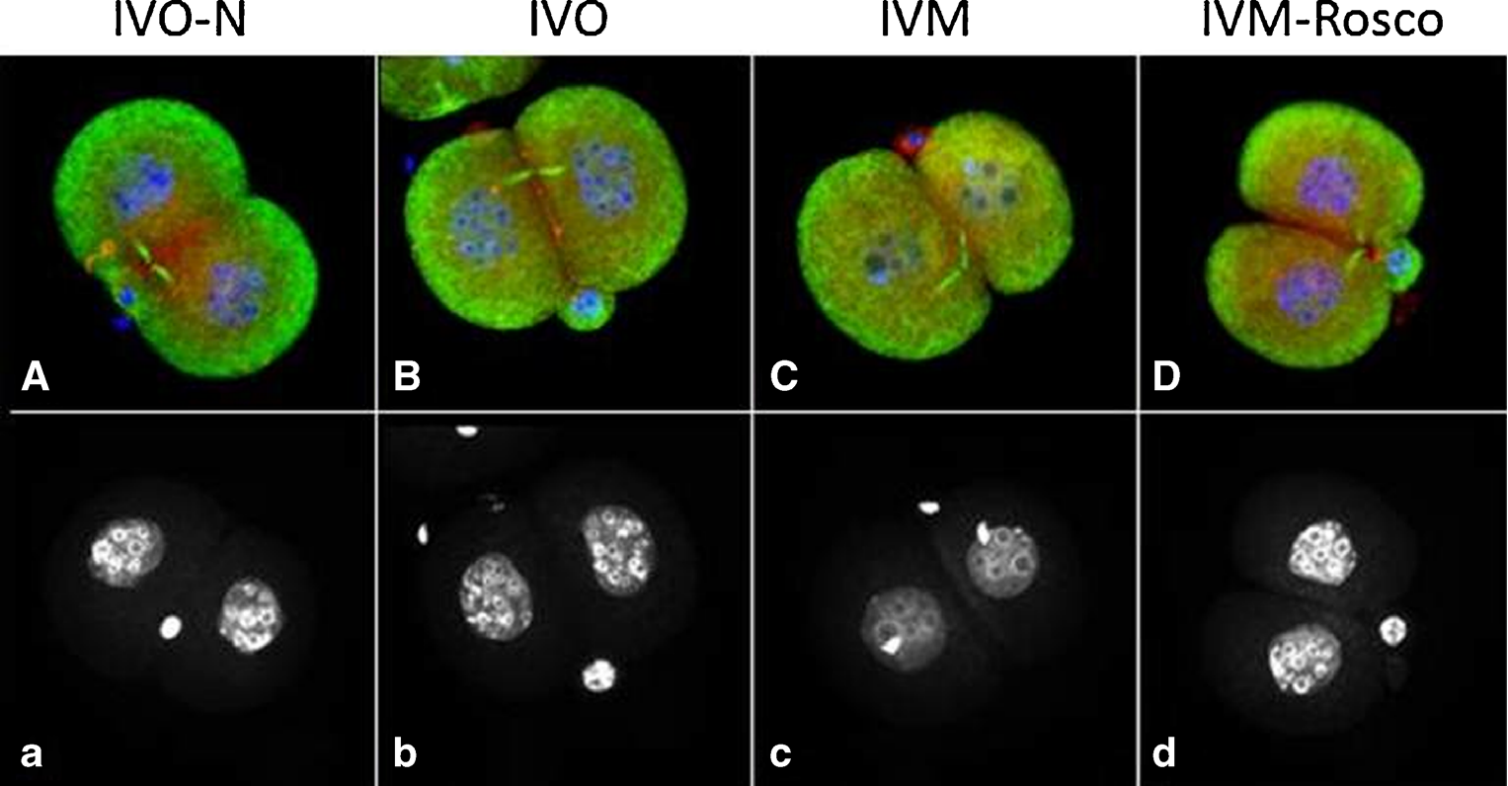
2-cell stage IVO-N, IVO, IVM and IVM-Rosco embryos labeled for tubulin (*green*), pericentrin (*red*) and chromatin (*blue*) (**A**–**D**), corresponding chromatin staining (**a**–**d**) depicting the number of small nucleoli on each blastomere. Note the asymmetric size of the two blastomeres of IVM 2-cell stage embryos (**C**) when compared to IVO (**B**) and IVM-Rosco (**D**). Also note that IVM 2-cell stage embryos (**c**) exhibit fewer nucleoli when compared to both IVO (**b**) and IVM-Rosco embryos (**d**), probably denoting distinct transcriptional activity

When embryonic morphology was analyzed, several features were observed to differ when IVO, IVM and IVM-Rosco-derived embryos were compared. As shown in Table 2, when 2-cell stage embryos were analyzed control-mating (IVO-N) and IVO derived embryos exhibited a symmetric first cleavage plan (94.4 and 71.4 %, respectively). However, IVM-derived 2-cell stage embryos exhibited increased asymmetries (84.8 %, Table 2, Fig. 3A–D) when compared to the same stage embryos of IVO-N, IVO and IVM-Rosco groups. Interestingly, IVM-Rosco derived 2-cell stage embryos displayed a more symmetric pattern of first cleavage (61.5 % symmetric versus 38.5 % asymmetric) that more closely resembled what was observed in IVO 2-cell stage embryos.

**Table 2:** Cleavage plan symmetries in 2-cell stage embryos

	Symmetric (%)	Assymetric (%)
Control-mating (*n* = 18)	94.4	5.6
IVO (*n* = 28)	71.4	28.6
IVM (*n* = 33)	15.2	84.8
IVM-ROSCO (*n* = 26)	61.5	38.5

Another feature we analyzed was the number of nucleolar-precursor bodies (NPBs) present at the 2-cell stage embryo. IVM-derived 2 cell stage embryos exhibited only 5–6 NPBs in each blastomere (*n* = 18 embryos) contrasting with the 16–17 NPBs present in the IVO group (*n* = 18 embryos). Interestingly, IVM-Rosco 2-cell stage embryos exhibited an intermediate number of NPBs in each blastomere (9–10, *n* = 10 embryos). Representative examples of this feature are shown in Fig. 3.

### In vitro maturation affects cell number at the blastocyst stage

**Fig. 4:**
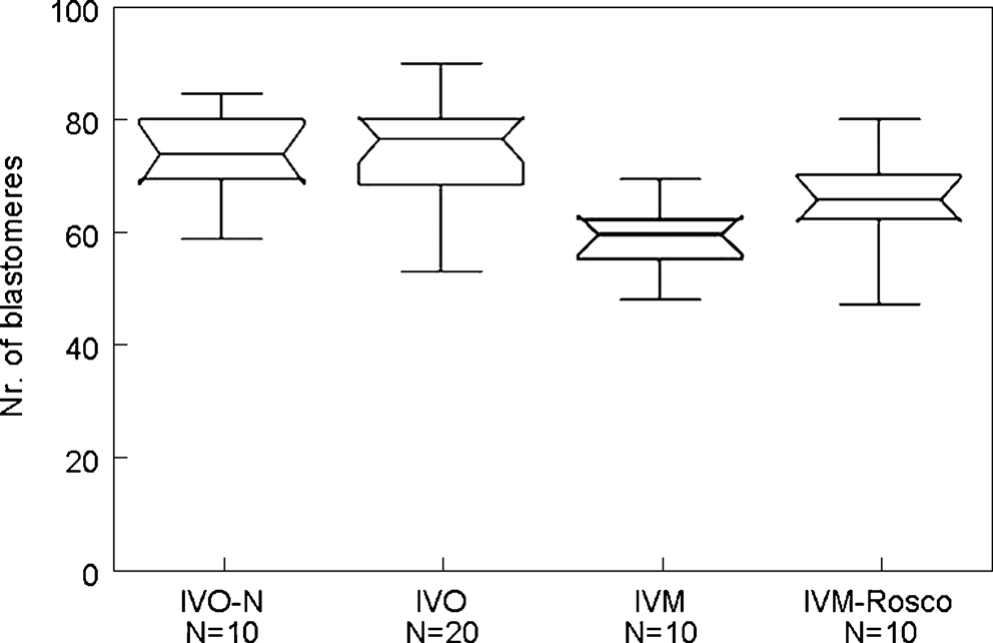
Total cell counts of IVO-N (control), IVO, IVM and IVM-Rosco blastocysts. Note the significantly less number of cells on the IVM blastocysts compared with both IVO-N and IVO. IVM-Rosco blastocysts exhibit an intermediate phenotype between IVM and IVO embryos

Total number of cells at the blastocyst stage was evaluated at day 5 based on the Hoechst 33258 nuclei staining. As shown in Fig. 4, IVO-derived blastocysts exhibited comparable cell numbers with the blastocysts produced from control-mating and analyzed at day 5 (73.35 ± 12,43 in IVO versus 72.3 ± 9.26 control-mating). In contrast, IVM-derived blastocysts display a low number of cells (56 ± 7.22). Interestingly, IVM-Rosco-derived blastocysts exhibit an intermediate number of blastomeres between IVO and IVM-embryos (63.4 ± 9.82).

### Centrosomal-microtubule organization of cleavage and blastocyst stage embryos

**Fig. 5:**
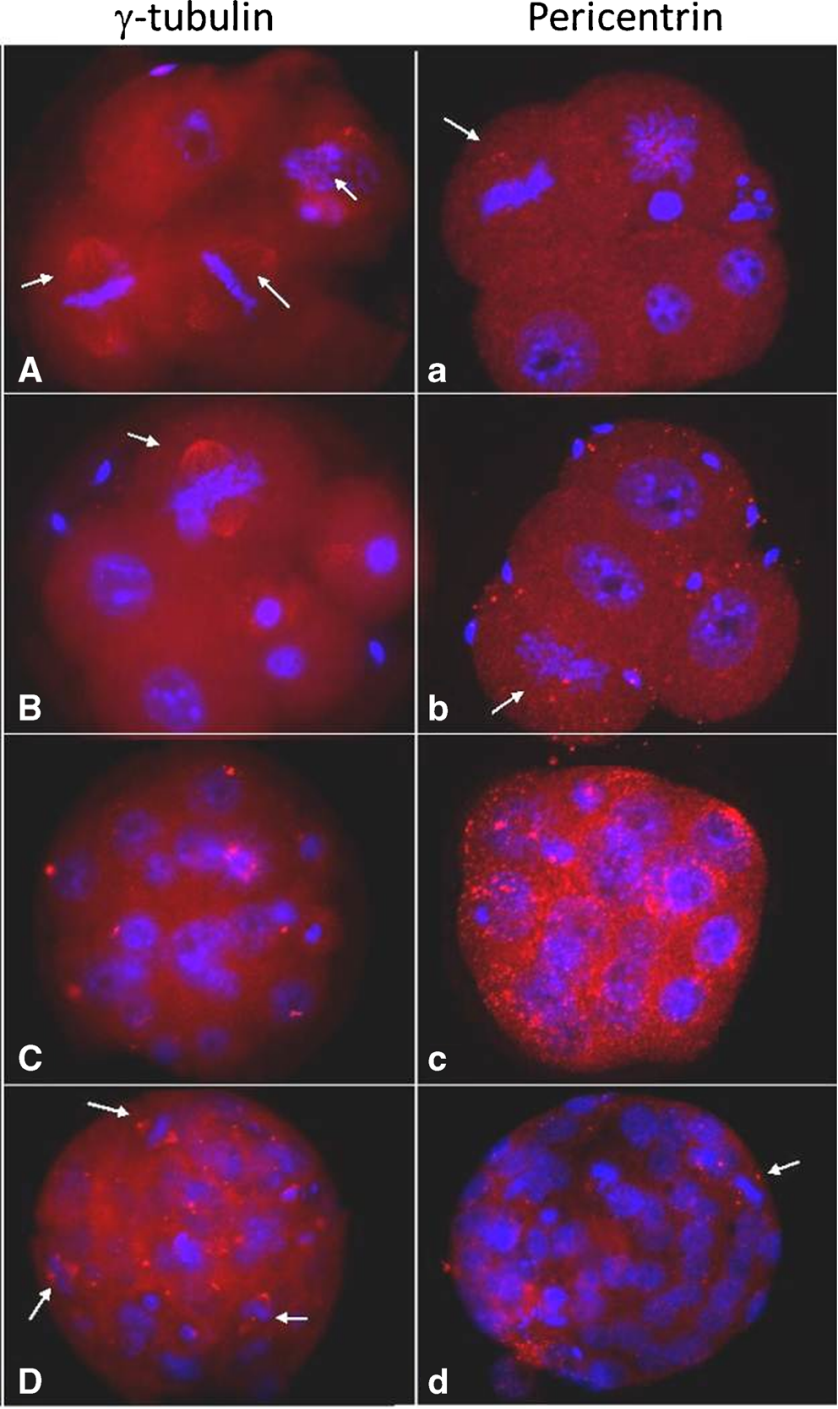
γ-tubulin (**A**–**D**) and pericentrin (**a**–**d**) pattern in mouse embryos. Chromatin is shown in *blue* (Hoechst staining). Note distribution of γ-tubulin (**A**, **B**), not pericentrin (**a, b**), forming the spindle during the first cleavages. γ-tubulin exhibits a punctuate perinuclear localization at compacted morula (**C**) and forms the spindle proper in blastocysts stage embryos (**D**). Pericentrin exhibits an apical distribution in 8-cell stage embryos (**c**) and forms the spindle poles in the blastocyst stage (**d**). *Arrow* indicate cell in metaphase

Since IVO, IVM and IVM-Rosco M-II oocytes were previously shown to exhibit distinct cytoskeleton organization [14, 15], we next examined distribution of the centrosomal-microtubule complex among the embryos produced. However, although the oocytes exhibited distinct cytoskeleton organization, these differences were not evident in the embryos produced. When microtubule, nuclear lamina organization and centrosomal distribution were evaluated no differences were observed between IVO, IVM and IVM-Rosco derived embryos. However, when pericentrin and γ-tubulin distribution was evaluated, a distinct organization of these centrosomal markers was observed in all groups as shown in Fig. 5.

**Fig. 6:**
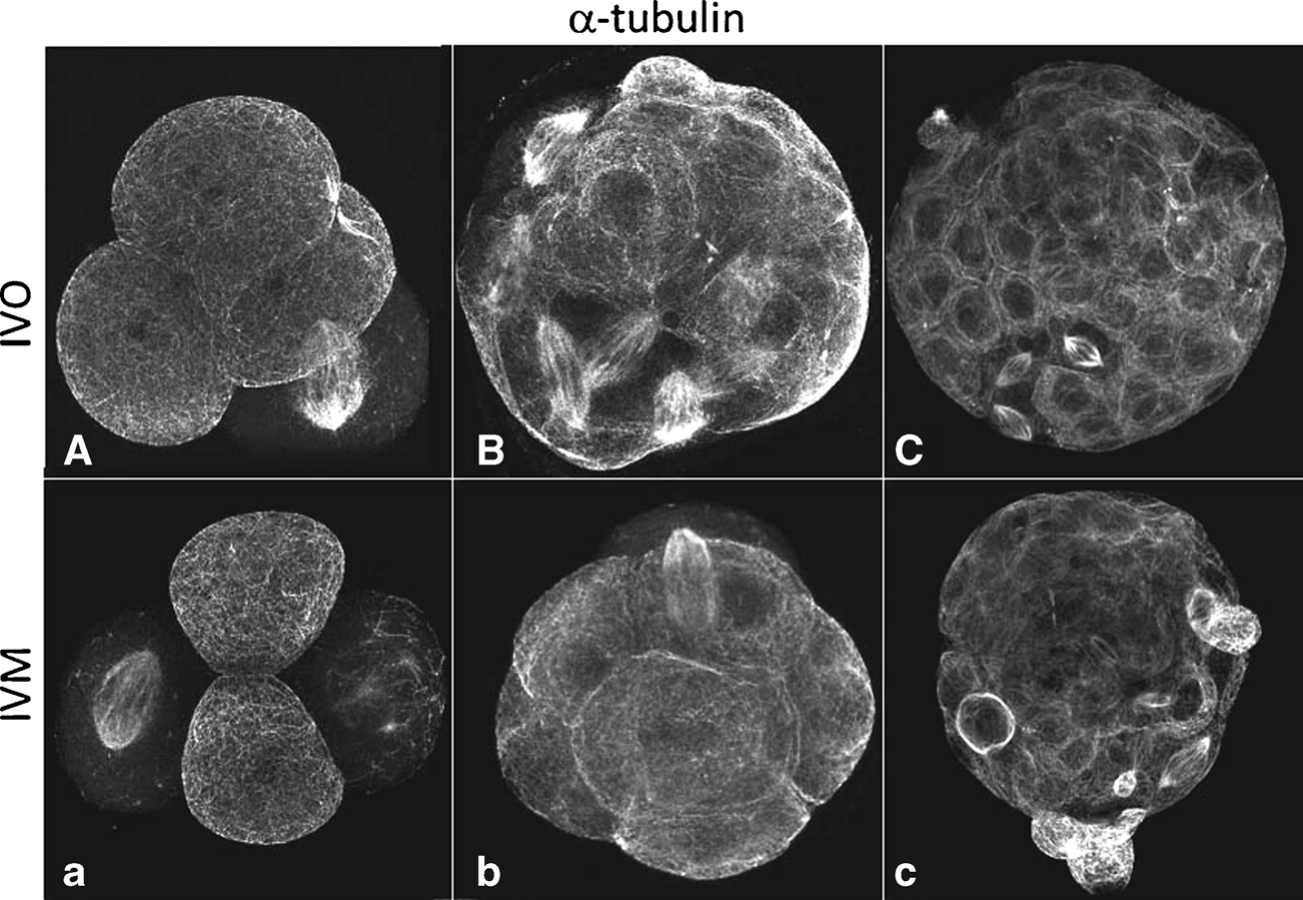
IVO (**A**–**C**) and IVM (**a**–**c**) embryos stained for α-tubulin evidencing pointed shape spindles. 4 cell-embryo (**A**,**a**), compacted morula (**B**, **b**) and blastocyst (**C**,**c**)

Pericentrin exhibits a dispersed cytoplasmic organization during the first cleavage stages until compaction (occurring between 8 and 16-cell stage embryo) where it starts showing a dispersed but apical distribution (Fig. 5c). In contrast, although γ-tubulin also exhibits a dispersed cytoplasmic distribution during the first cleavage stages, it starts showing punctuate and perinuclear staining in compacted stage embryos (Fig. 5C). Interestingly, whenever a spindle is observed γ-tubulin is distributed throughout the spindle (Fig. 5A, B). In contrast, pericentrin is only observed at the spindle poles (Fig. 5a,b). This differential distribution of γ-tubulin and pericentrin was noted until the blastocyst stage with γ-tubulin distributed throughout the spindle and pericentrin restricted to the spindle poles (Fig. 5D and d, respectively). Regardless of the embryonic stage, blastomeres exhibit pointed-shape spindles that assume a very focused appearance at the blastocyst stage as shown in Fig. 6.

**Fig. 7:**
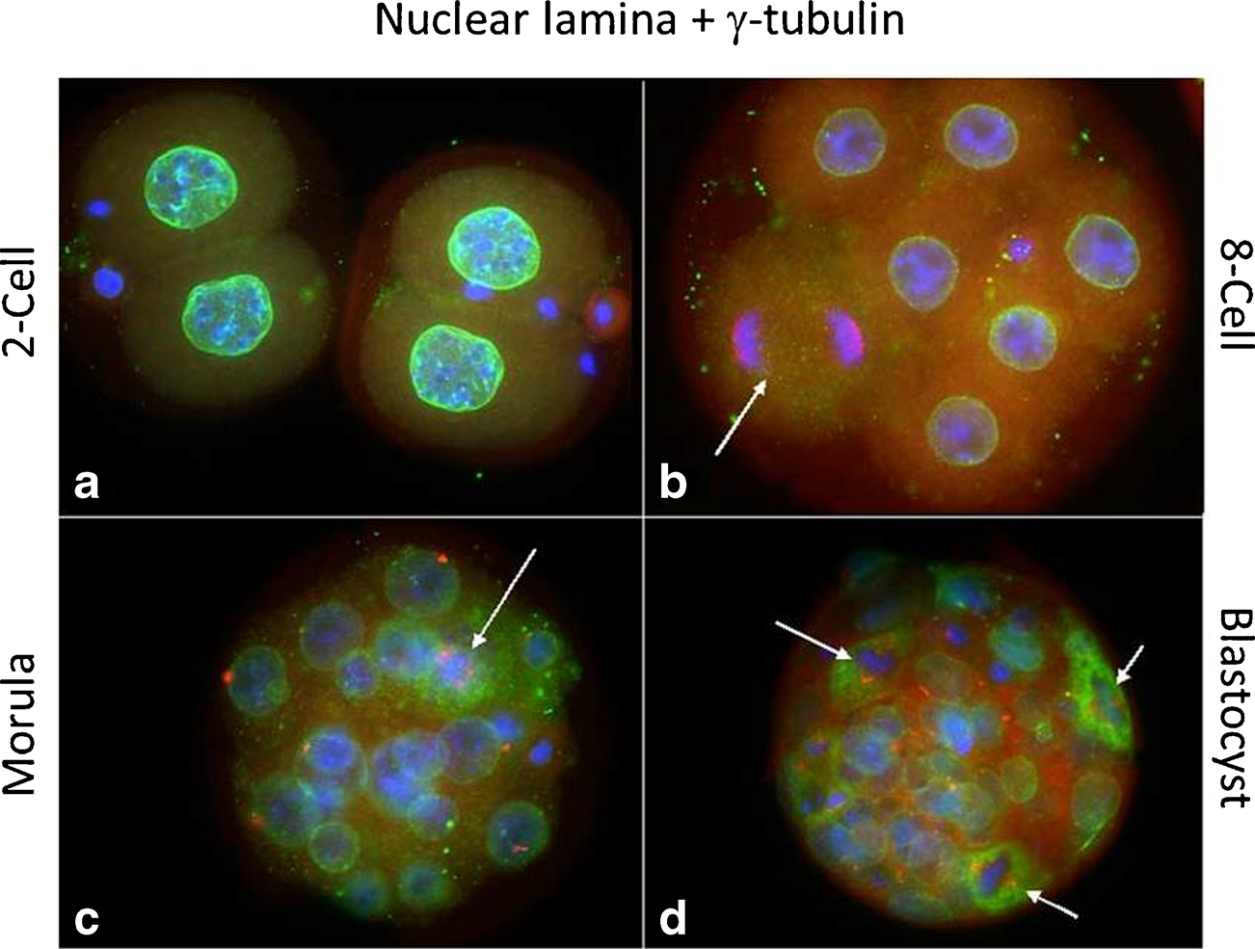
2-cell embryo (**a**), 8-cell embryo (**b**), compacted morula (**c**) and blastocyst (**d**) depicting nuclear lamina (lamin B, *green*), γ-tubulin (*red*) and chromatin (Hoechst 33258, *blue*). Note that nuclear lamina is dispersed in the cytoplasm in dividing cells (*arrows*)

Nuclear lamina distribution, as expected, always exhibited a regular pattern outlining the nucleus of each blastomere or, whenever a spindle was observed, dispersed through the cytoplasm of each dividing blastomere (Fig. 7).

## Discussion

In the present study, the developmental competence of IVM and IVM-Rosco oocytes was directly compared with IVO oocytes. The hypothesis that the distinct cytoskeleton configurations found in IVO, IVM and IVM-Rosco oocytes reflects distinct pre-implantation developmental competence was addressed [14–16]. To deal with this question we have performed IVF and assessed subsequent pre-implantation development. The embryos produced were daily observed, cleavage rates assessed, blastomere size evaluated and the total number of cells counted at the blastocyst stage. Additionally, to characterize cytoskeleton organization of the embryos produced, embryos were fixed and stained for evaluation of microtubule patterning, regulative (γ-tubulin) and constitutive (pericentrin) centrosomal markers, and nuclear lamina distribution. Our results show that IVM oocytes, as predicted, impaired fertilization efficiency, compromise developmental competence, and reveal abnormalities in blastomere symmetry and in nuclear morphology throughout cleavage and until the blastocyst stage.

Interestingly, IVM-Rosco-derived embryos show less dramatic perturbations, bringing it closer to the IVO-derived embryo characteristics, suggesting that the unique properties that underlie coordination of nuclear and cytoplasmic events during oocyte maturation may be preserved with the use of a cell cycle arresting agent during oocyte maturation.

### Both groups of IVM-derived embryos exhibit low fertilization efficiencies

We have previously shown that M-II IVM oocytes exhibit loosen barrel-shaped spindles contrasting with the compacted pointed-shape spindles, cortically localized, of IVO oocytes [14, 15, 25]. Additionally, when compared to IVO spindles, IVM spindles exhibited decreased phosphorylation levels detected by the mitotic phosphoprotein antibody MPM-2 [14]. Since important cell cycle factors involved in egg activation and pronuclear formation were found to be localized to the spindle, especially to the spindle poles [26–28] a miss-localization of these factors could have deleterious implications in egg activation and fertilization efficiency. Furthermore, depletion of MTOC material from the cytoplasm was found to occur in IVM oocytes [14, 15], and this depletion predicted to increase with second polar body emission at fertilization [16]. Since maternal γ-tubulin has been shown to be critical for microtubule reorganization during fertilization and pronuclear apposition [29], it was expected that IVM oocytes, which are depleted from γ-tubulin stores in their cytoplasm, would exhibit impaired fertilization efficiency. Although IVM-Rosco M-II oocytes have been shown to exhibit increased number of cytoplasmic MTOCs, and cortically localized pointed-shape spindles [15] their fertilization efficiency was also low. These results may imply that the described configurations have a longer term-effect seen only when analyzing pre-implantation development. However, it could also be linked to a negative effect of the drug on the ability of the oocyte to be fertilized, as it has been previously described in porcine oocytes [30]. However, further studies are needed to determine the exact impact of this drug on the developmental competence of matured mouse oocytes.

### IVM directly impacts pre-implantation development

The dynamics of the preimplantation embryo cell cycle, specifically the timing and pace of the first embryonic divisions has been correlated with the developmental potential of human embryos [31–33]. Since the number of cytoplasmic MTOCs in the oocyte was considered an important maternal factor necessary to support first embryonic cleavages [16, 34–36] and given that IVO, IVM and IVM-Rosco oocytes displayed characteristically distinct number of cytoplasmic MTOCs [14, 15], we further analyzed cleavage rates of the embryos produced.

When cleavage rates were compared between these groups, IVO oocytes exhibited synchronous and efficient developmental progression until the blastocyst stage while IVM and IVM-Rosco oocytes displayed a delayed progression until the blastocyst stage. This delay was clearer in IVM than in IVM-Rosco embryos, both at day 4 and 5 depicting deficient cell cycle progression. IVM-Rosco embryos exhibited a more effective cell cycle progression probably due to a spatial organization of their microtubule-centrosomal complex closer to the IVO situation.

In addition, IVM-Rosco blastocysts exhibited higher number of cells at the blastocyst stage when compared to IVM oocytes. If the maternal MTOC legacy is an important factor to support cell cycle progression during preimplantation development [35, 37], it would be expected that IVM-Rosco oocytes once fertilized exhibited increased cell cycle progression during preimplantation development when compared to their IVM counterparts. Indeed, not only is the total cell number in the blastocyst stage higher, but also developmental progression until the blastocyst stage seems more efficient in IVM-Rosco embryos than in IVM embryos.

One of the major events during preimplantation development is the maternal to zygotic transition that, in the mouse, occurs at the 2-cell stage when important embryonic genes are switched on [38]. When 2-cell stage embryos were analyzed, IVM embryos exhibited distinct numbers of nucleoli precursor bodies (NPBs) when compared to IVO embryos. NPBs were found to contain the major proteins for rRNA synthesis [39, 40]. Since IVM embryos exhibit less NPBs than IVO or IVM-Rosco 2-cell stage embryos, and although further studies are needed to prove this hypothesis, we speculate that abnormalities exist in genome activation and ultimately nuclear function in IVM embryos.

Actin cytoskeleton has been implicated in cell polarity, chromosome migration and asymmetric divisions during oocyte maturation [41–43]. An intact actin network has also been responsible for the symmetric cell divisions of the mouse oocyte [44]. IVM-derived 2-cell embryos exhibited asymmetrical first cleavages denoting a loss of spatial control of cytokinesis after PN apposition. The same did not happen with IVO and IVM-Rosco 2-cell stage embryos where the appearance of a symmetric first cleavage plan was observed. A deficiency in the first cleavage plan site that occurs upon fertilization originates asymmetric blastomeres and may compromise development to the blastocyst stage, as is well known in the human [44, 45]. Unequal distribution of important cell cycle regulators after the first cleavage might affect subsequent development of the embryo [46].

Despite the involvement of the cortical microfilaments, what exactly defines the place where the first cleavage plane forms remains a controversial topic [45, 47]. However, it is interesting to correlate the distinct spatial organization of the centrosome-microtubule complex in IVM M-II oocytes to a loss of spatial polarity during M-II arrest [16]. Our results suggest that delaying the onset of cell cycle progression during oocyte maturation has a benefit effect in terms of cortex remodeling culminating in symmetric cleavage upon fertilization of IVM-Rosco-derived embryos.

Collectively, the present study showed that fertilization efficiency, pre-implantation developmental progression, blastomere division symmetries, blastomere nuclear morphology and blastomere cell number at the blastocyst stage are impacted in embryos produced from IVM oocytes. Manipulations during the early stages of IVM aimed to arrest cell cycle progression were partially effective in correcting these perturbations highlighting the potential utility of cell cycle synchronizing agents like roscovitine. Given the growing emphasis on time-lapse microscopy as a prognostic tool for embryo quality evaluation, the present study identifies novel embryonic cytoskeletal biomarkers that take their origin from conditions used during in vitro oocyte maturation. We suggest that these distinct cytoskeleton characteristics may be considered as additional biomarkers for mouse oocyte developmental quality. Further studies are needed in tractable experimental rodent and non-human primate and human oocytes to assess the value of using cell cycle arresting drugs during IVM protocols. If benefits upon developmental competence of IVM oocytes are confirmed, this will surely have a strong impact upon current human ART IVM procedures and outcomes.
